# Patient-friendly integrated first trimester screening by NIPT and fetal anomaly scan

**DOI:** 10.1186/s13039-020-00525-y

**Published:** 2021-01-09

**Authors:** Malgorzata Ilona Srebniak, Maarten F. C. M. Knapen, Marieke Joosten, Karin E. M. Diderich, Sander Galjaard, Diane Van Opstal

**Affiliations:** 1 Department of Clinical Genetics, Erasmus MC, Wytemaweg 80, 3015 CN Rotterdam, The Netherlands; 2 Department of Obstetrics and Fetal Medicine, Erasmus MC, Rotterdam, The Netherlands

**Keywords:** NIPT, Fetal anomaly scan, First trimester, Prenatal screening

## Abstract

Many major structural fetal anomalies can be diagnosed by first trimester fetal anomaly scan. NIPT can accurately detect aneuploidies and large chromosomal aberrations in cfDNA in maternal blood plasma. This study shows how a patient-friendly first trimester screening for both chromosomal and structural fetal anomalies in only two outpatient visits can be provided. Genotype-first approach assures not only the earliest diagnosis of trisomy 21 (the most prevalent chromosome aberration), but also completion of the screening at 12–14 weeks. To ensure proper management and avoid unnecessary anxiety abnormal NIPT different from trisomy 21, 18 and 13 should be referred for genetic counseling.

First trimester prenatal screening for fetal chromosomal aberrations is nowadays challenged by different testing options [1]. For more than a decade, the first trimester combined test (CT) including Nuchal Translucency (NT) measurement was the most sensitive screening test [2], but in some countries it is nearly completely replaced by the non-invasive prenatal test (NIPT) [3]. This means that at this moment in the Netherlands in the majority of pregnancies early fetal ultrasound only includes a dating-scan. This scan is generally performed around the 10th gestational week and it is limited to the confirmation of fetal viability, determination of gestational age, and in case of a multiple pregnancy, to determination of the number of viable fetuses and chorionicity. If no apparent major fetal anomaly is noticed during this dating scan, current policy is to offer NIPT or CT from the 11th gestational week on [3], however only a small minority of patients chooses CT. In case of apparent ultrasound anomalies or a NT ≥ 3.5 mm an invasive diagnostic test is offered instead of NIPT.

The implementation of a first trimester fetal anomaly scan (FAS) [4–7] for all pregnant women is due in the near future in the Netherlands. When integrating a first trimester FAS into the Dutch prenatal screening program, several scenarios are possible. Since NIPT can be performed from the 10th gestational week, it may precede the FAS. Offering NIPT only when the FAS is uneventful might be another option as well to prevent confusion for pregnant women due to the different testing options. A simple, straightforward clinical scenario is needed, in which accessibility and logical consecutive investigations with the lowest burden for the patients are key-factors. It should also allow early screening and diagnosis, important for both patients and the health care system. In our experience a pregnant woman prefers a diagnosis as early as possible to achieve early reproductive options and awareness on favorable results. And most patients, who choose for either kind of genetic testing (NIPT or microarray), prefer genome-wide analysis [3, 8]. So we think that many of them will opt for both NIPT and a first trimester FAS. Early screening has a limited time window between 10 and 14 weeks of gestation, therefore the priorities in this proposal are:

1. as early as possible diagnosis of chromosomal and non-chromosomal anomalies,
2. implementation into the routine primary pregnancy care and,
3. completion within only two patient visits.

## Genotyping first or phenotyping first?

**Fig. 1:**
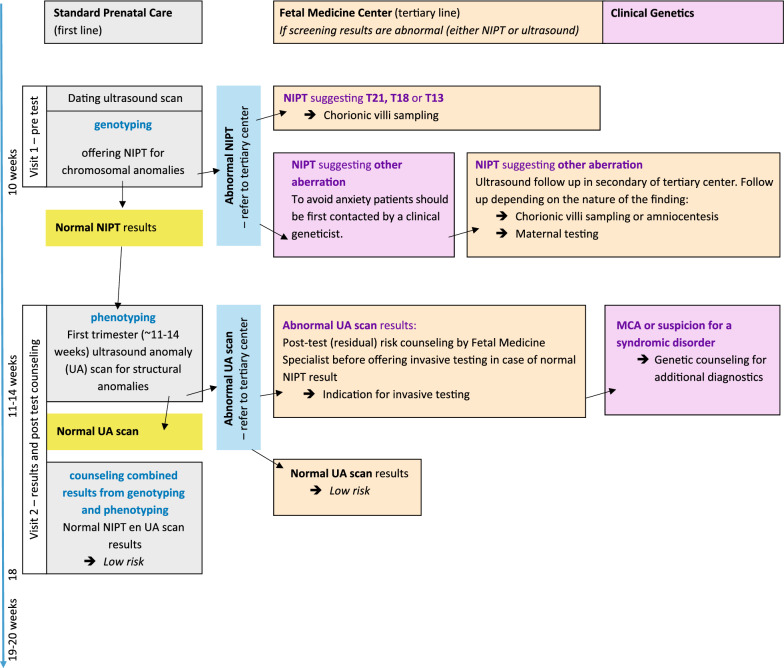
Integrating NIPT and fetal anomaly scan for efficient and patient-friendly first trimester screening. *MCA* multiple congenital anomalies

When taking the issues mentioned above into account, the most important question that arises is whether to perform genotyping or phenotyping as the starting exam. When a patient wishes the earliest possible diagnosis, then NIPT, performed from 10 weeks of gestation, would be the first test (Fig. 1) in consecutive line. The Dutch TRIDENT studies showed a mean reporting time of ~ 6 days, and since the results of chorionic villi sampling (CVS) can be achieved within 1–2 weeks, a final diagnosis is possible between 12 and 13 weeks gestational age. Moreover, the TRIDENT studies showed that the overall technical NIPT failure rate in the Dutch laboratories is low: 0.3% among samples taken from 10 weeks of gestation [9] and 0.2% (161/73.239) among samples taken from 11 weeks of gestation [3]. Also, the proportion of false negative results probably due to a low fetal fraction (FF) or mosaicism is very low (0.01%—10 cases out of 73 239 [3]). Therefore, in this setting there is no argument to delay NIPT testing after 13–14 weeks for technical reasons. This is confirmed by Wang et al. [10] who showed that the FF raises only 0.1% per week between 10 and 20 weeks of gestation.

The Dutch TRIDENT 2 study showed that the majority of patients after normal dating scan will receive normal NIPT results who can then proceed to the FAS. In total only 0.75% (550/73239) of patients received abnormal NIPT results, either for trisomy 13/18/21 (343) or other findings (207), which requires referral for invasive testing (see Fig. 1). So far the uptake of NIPT in the Netherlands is moderate, however, it is expected to significantly increase if it could be reimbursed similar to screening by ultrasound [11, 12]. In Dutch settings the expectation is that after subsequent FAS, about 0.2–0.3% of patients with normal NIPT results will show abnormal FAS outcome [6]. Reiff and colleagues found 0.4% structural anomalies or cystic hygroma after normal NIPT results (2.1% if NT > 3 mm was included) [13]. Therefore we assess that performing the ultrasound scan before NIPT will not lead to substantial cost-effectiveness. Both NIPT and early ultrasound detect comparable percentage of aberrant cases and each component has an important impact, but cannot fully substitute the other. Moreover we believe that performing NIPT after FAS will delay the prenatal diagnosis of fetal aneuploidies and other chromosomal aberrations detectable by NIPT.

## The first visit

This first visit in pregnancy (in the Netherlands mostly at a community midwife (primary care)) could include the dating scan as well as offering pre-test counseling for integrated prenatal screening (in our proposal: both for NIPT and FAS), followed by a blood sampling if the patient chooses to proceed.

Before the second visit, NIPT results are expected to be ready:

- When the NIPT results show an increased risk for trisomy 13, 18 and 21, the patient should be referred for invasive testing (Fig. 1) and a CVS can be offered to ensure rapid diagnosis [14].
- Other abnormal NIPT findings should be referred for genetic counseling by a dedicated clinical geneticist, who closely collaborates with fetal medicine specialists, to ensure proper pregnancy management and avoid unnecessary anxiety. Appropriate follow up testing will depend on the chromosome aberration involved [14] and whether the aberration may have a maternal or fetal origin.
- In case of normal results: the patient does not need to be contacted before the second visit.

## The second visit: first trimester FAS and post-test normal NIPT counseling

To make patient’s care pragmatic and lower the social-economic and health care burden we suggest to integrate post-test counseling on normal NIPT results with post-test counseling after first trimester FAS (Fig. 1).

When NIPT shows a normal result, but the subsequent first trimester FAS shows a structural abnormality, the patient needs to be referred for an expert ultrasound scan. When the fetal anomaly is confirmed, the post-test counseling needs to concentrate on the residual genetic risks: both the risk for a false negative NIPT result and the risk for genetic anomalies that cannot be detected by current NIPT approaches. Invasive testing should be offered. In case of multiple congenital anomalies or a suspected genetic syndrome patients should subsequently be referred for genetic counseling (according to the current standards).

## Confirmatory diagnostic testing by CVS

The use of amniocentesis for confirmation of abnormal NIPT performed early in pregnancy (at 10–11 weeks of gestation) may lead to an unacceptable long waiting time for the prospective parents, since amniocentesis can only be safely performed after 15 weeks of gestation. Therefore we previously explored whether CVS, that can be performed in the first trimester of pregnancy, could be a reliable alternative for an amniocentesis. It is often believed that CVS and NIPT investigate the same placental tissue, and that CVS for NIPT confirmation should be avoided. However, whereas NIPT investigates the cytotrophoblast of CV, with CVS, another cell lineage is investigated, namely the mesenchymal core of CV, which has the same embryonic origin as the fetus itself (the inner cell mass). Therefore, the latter better reflects the chromosomal status of the fetus than the cytotrophoblast, which originates from the embryonic trophoblast. However, there is a small risk for detecting chromosomal mosaicism in the mesenchymal core that potentially is confined to the placenta and not present in the fetus (confined placental mosaicism, CPM). This will require follow-up diagnostic testing of amniotic fluid to differentiate between CPM and generalized mosaicism that also affects the fetus. This is a limitation of CVS that always should be considered during pre-test counseling for any indication. Based on our experience with cytogenetic investigations in CV and on the literature, we have previously shown [14], that in the majority of cases (97%) CVS can reliably be used for confirmation of NIPT positive for trisomy 13, 18, and 21 [14], despite the phenomenon of CPM [15]. The risk for a second invasive procedure is the smallest in case of trisomy 21 (1.6%), intermediate in case of trisomy 18 (3.2%) and the highest in case of trisomy 13 (8.3%). This was confirmed by Grati et al. [16] in a large series of CVS. In cases in which NIPT shows an increased risk for trisomy 13, early ultrasound may aid the decision on CVS versus amniocentesis.

For other autosomal trisomies that may be detected with genome wide NIPT testing, the type of confirmatory test (CVS or amniocentesis) will depend on the chromosome aberration involved. Of course also other factors such as patient preferences, the presence of fetal ultrasound anomalies and gestational age will guide the choice of invasive procedure [14, 16, 17].

## Conclusions

According to this proposal the majority of patients (~ 98%) with most favorable results, where both NIPT and first trimester FAS are normal, will have completed the first trimester screening at 11–14 weeks. Early prenatal screening and counseling for both chromosomal and major fetal structural anomalies will be completed within two outpatient visits. These patients can then be offered routine second trimester FAS. Our two step early screening proposal combines the aim of early diagnosis, offering women early and safe therapy options, together with a screening program expected to be effective, efficient and patient-friendly.

## Data Availability

Not applicable.

## Abbreviations

CT: Combined test
CVS: Chorionic villi sampling
FAS: Fetal anomaly scan
MCA: Multiple congenital anomalies
NIPT: Non-invasive prenatal test
NT: Nuchal translucency
